# Development of an Electrochemical Platform Based on Zinc Oxide Nanoparticles Embedded onto Montmorillonite Clay Functionalized with Phenylalanine for the Nano-Sensing of Acetaminophen in Pharmaceutical Tablets

**DOI:** 10.3390/bios16050244

**Published:** 2026-04-26

**Authors:** Gildas Calice Wabo, Alex Vincent Somba, Sengor Gabou Fogang, Cyrille Ghislain Fotsop, Astree Lottie Djuffo Yemene, Léopoldine Sonfack Guenang, Marcel Cédric Deussi Ngaha, Gullit Deffo, Evangeline Njanja

**Affiliations:** 1 Electrochemistry and Chemistry of Materials, Department of Chemistry, Faculty of Sciences, University of Dschang, Dschang P.O. Box 67, Cameroon; gildas.wabo@univ-dschang.org (G.C.W.); vincent.somba@univ-dschang.org (A.V.S.); sengor.gabou@univ-dschang.org (S.G.F.); lottie.djuffo@univ-dschang.org (A.L.D.Y.); guenang.sonfack@ubuea.cm (L.S.G.); 2 Institute of Chemistry, Faculty of Process and Systems Engineering, Universität Platz 2, 39106 Magdeburg, Germany; fotsopcyril@yahoo.fr; 3 Department of Chemistry, Higher Teacher Training College, The University of Bamenda, Bamenda P.O. Box 39, Cameroon; marcel_cedric.ngaha_deussi@univ-fcomte.fr

**Keywords:** acetaminophen, ZnO nanoparticles, montmorillonite clay, phenylalanine, electrochemical sensor

## Abstract

This study describes the development of an electrochemical sensor for quantitatively measuring acetaminophen (ACOP) in drug tablets. The sensor design is based on the modification of glassy carbon electrode (GCE) using zinc oxide nanoparticles (ZnONPs) embedded in a naturally occurring clay matrix (Sa) functionalized with phenylalanine (Phe). To ensure that the ZnONPs are homogeneously dispersed on the clay surface, the nanocomposite was synthesized using an impregnation approach and low-temperature heat treatment. The amino acid promotes specific interactions with ACOP through hydrogen bonding and π-π stacking, acting as both a stabilizing agent and a molecular recognition moiety. FTIR, UV-Vis, XRD, and FESEM/EDX mapping were employed to fully characterize the developed material (ZnONPs-Sa/Phe). Cyclic voltammetry (CV) and differential pulse voltammetry (DPV) were used for the electrochemical determination of ACOP using the modified electrode GCE/ZnONPs-Sa/Phe. Parameters susceptible to affecting the sensitivity of the developed sensor were optimized, revealing that 5 µL of the suspension ZnONPs-Sa/Phe immobilized on GCE was ideal for the sensing of ACOP in a phosphate buffer solution at pH 2.0. The calibration curve obtained by plotting peak current intensity against ACOP concentration exhibited linear behavior within the concentration range between 0.02 µM and 0.28 µM, enabling determination of the limits of detection (LOD) and quantitation (LOQ) at 8.54 × 10^−9^ M and 2.84 × 10^−8^ M, respectively. The reproducibility, stability, and selectivity of the sensor were evaluated, followed by its application to the nano-sensing of ACOP in Africure and Doliprane tablets, yielding satisfactory results. The simplicity, affordability, and high analytical sensitivity of the developed sensor make this sensing platform a promising tool for pharmaceutical quality control applications.

## 1. Introduction

Quantitative analysis of pharmaceutical agents is a key component of analytical chemistry and is essential to ensuring the efficacy, safety, and uniformity of pharmaceutical formulations. There is urgent need for analytical methods that are fast, sensitive, and economically feasible in response to the growing pharmaceutical industry [1]. Paracetamol (also known as acetaminophen, ACOP) is one of the most commonly used analgesic and antipyretic medications due to its excellent tolerability, high therapeutic efficacy, and low cost [2]. However, consuming more than the recommended daily dose of 4 g has been linked to serious toxicological consequences, such as nephrotoxicity, hepatotoxicity, and cardiovascular problems [3]. Therefore, both clinical monitoring and regulatory quality control depend on precise, trustworthy measurements of paracetamol in pharmaceutical formulations and biological matrices.

ACOP has been determined using various analytical techniques, including liquid chromatography [4], UV-visible spectrophotometry [5], chemiluminescence [6], and high-performance liquid chromatography [7]. While these methods are renowned for their precision, they are often constrained by the need for specialized equipment, intricate sample preparation, substantial reagent usage, and reliance on highly skilled personnel. Such limitations can hinder their feasibility for routine and large-scale analyses [8,9]. Due to their inherent advantages, including high sensitivity, ease of use, low reagent and sample consumption, potential for miniaturization, and suitability for in situ and real-time monitoring, electrochemical sensing techniques have emerged as appealing alternatives to traditional analytical methods [8,10,11,12]. However, pharmaceutical and biological matrices are often highly complex, containing low concentrations of target analytes that can interfere with other chemicals, making them difficult to detect. Consequently, current research focuses on enhancing sensor performance in terms of analytical reliability, selectivity, and sensitivity. A popular strategy for enhancing sensor performance is to improve the reactivity of the conventional working electrodes, such as glassy carbon electrode (GCE), by immobilizing functional materials such as polymers, amino acids, mesoporous structures, and nanomaterials on their surfaces. For instance, Yanalak et al. (2021) reported the used of a ternary nanocomposite of mesoporous graphitic carbon nitride/black phosphorus/gold nanoparticles for the photocatalytic hydrogen evolution and electrochemical sensing of paracetamol, leading to a limit of detection (LOD) of 0.042 µM [13]. More recently, Vomo et al. (2024) performed the synthesis of zinc oxide nanoparticles based on coffee husks embedded on mesoporous silica for the sensing of acetaminophen, leading to an LOD of 0.11 µM [14]. Several other electrochemical sensors of paracetamol have been developed [15,16,17], showing the current state of the art for the determination of ACOP in aqueous medium. In this context, nanomaterials are of particular interest due to their exceptional physicochemical properties, including high mechanical strength, electrocatalytic activity, large surface-to-volume ratio, superior electrical conductivity, and ease of functionalization [8,9,18,19]. In addition, clay minerals, particularly smectite clays such as montmorillonite, have shown considerably interest as potential sensor components thanks to their large specific surface area (typically 80–150 m^2^/g), high cation exchange capacity (up to 78.2 meq/100 g), structural resilience, and ability to intercalate various functional molecules [15,16,17]. Furthermore, the functionalization of clay matrices with zinc oxide nanoparticles (ZnONPs) greatly improves their electrochemical performance by increasing conductivity and catalytic efficiency. This is primarily due to ZnO’s high electron mobility and semiconducting properties, which accelerate the charge transfer processes at the electrode interface [20,21]. Additionally, the capacity for chemical recognition of the hybrid system is enhanced by the addition of amino acids such as phenylalanine (Phe). Phe improves the nanocomposite’s structural integrity and functional adaptability of the nanocomposite while promoting the selective binding of the target analytes through hydrogen bonding and π-π stacking interactions.

In this study, we present the development of a novel hybrid nanomaterial for the electrochemical sensing of acetaminophen. This hybrid nanocomposite comprises zinc oxide nanoparticles (ZnONPs), natural smectite clay (Sa), and phenylalanine (Phe), which are drop-coated on a bare GCE. The synergetic effects of ZnONPs, Sa, and Phe on the GCE/ZnONPs-Sa/Phe sensor are assumed to improve the selective adsorption of ACOP through molecular recognition, increase the mechanical and chemical robustness of the sensor surface, and enable effective charge transfer. A thorough analytical examination of the sensor showed excellent performance in identifying acetaminophen in pharmaceutical tablets, highlighting its potential use for pharmaceutical quality control.

## 2. Materials and Methods

### 2.1. Chemicals and Reagents

The reagents used were all analytical grade, purchased from reliable commercial vendors. For instance, acetaminophen (ACOP, C_8_H_9_NO_2_, 99%), ponceau 4R (C_20_H_11_N_2_Na_3_O_10_ S_3_, 99%), ciprofloxacin (C_17_H_18_FN_3_O_3_, 98%), tartrazine (C_8_H_14_ClN_5_, 99%), diclofenac (C_14_H_11_Cl_2_NO_2_, 99%), ethanol (C_2_H_5_OH, 99.9%), alumina (Al_2_O_3_, 98%), and zinc nitrate hexahydrate (Zn(NO)_2_·6H_2_O, 99%) were from Sigma-Aldrich (Taufkirchen, Germany). Additional chemicals, including potassium chloride (KCl, 99.5%), mercuric chloride (Hg_2_Cl_2_, 99%), cadmium nitrate tetrahydrate (Cd(NO_3_)_2_·4H_2_O, 98.5%), lead nitrate (Pb(NO_3_)_2_, 99%), boric acid (H_3_BO_3_, 98%), sodium hydroxide (NaOH, 99%), potassium dihydrogen phosphate (KH_2_PO_4_, 99%), dipotassium hydrogen phosphate (K_2_HPO_4_, 99%), sodium acetate (CH_3_COONa, 99%), acetic acid (CH_3_COOH, 98%), sodium chloride (NaCl, 98%), and phosphoric acid (H_3_PO_4_, 63%), were from Prolabo (Tokyo, Japan) or Fisher Scientific (Nidderau, Germany). The montmorillonite clay mineral utilized in this study, designated as Sa, was collected in Sabga, Tubah district, Mezam department of the northwest region of Cameroon. Its chemical formula is approximately NaSi_16_(Al_6_ FeMg)O_20_(OH)_4_ [22]. The extraction and purification procedure was reported in the previous work of our team [15,23,24,25].

### 2.2. Synthesis of Electrode Material (ZnONPs-Sa/Phe)

**Figure 1:**
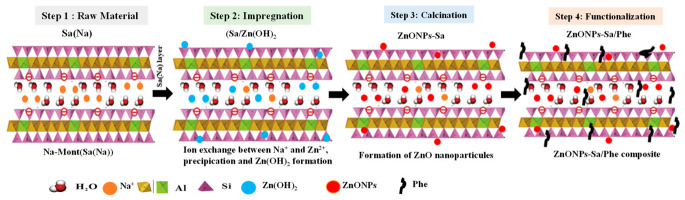
Step-by-step method for the synthesis of ZnONPs-Sa/Phe.

A modified process based on the method outlined [26] was adapted for the synthesis of zinc oxide nanoparticles (ZnONPs) impregnated onto raw clay. Briefly, 0.4 g of sodium hydroxide (NaOH) and 0.3 g of zinc nitrate pentahydrate (Zn(NO_3_)_2_·5H_2_O) were added to 10.125 mL of deionized water, and the mixture was stirred at 60 °C for about 10 min prior to adding 3.375 g of sodium montmorillonite clay (Sa(Na)). The mixture was transferred into an autoclave and heated at 50 °C for one hour to successfully extract the interlamellar water from the montmorillonite structure. The suspension was then poured into a three-necked flask and degassed with nitrogen under vacuum for 30 min at room temperature in order to remove any remaining air and promote the release of smectite pores. After two hours of constant stirring, the resulting suspension was centrifuged and the recovered product was dried in an oven at 70 °C and calcined at 500 °C for 5 h in a muffle furnace to promote the crystallization and the strong binding of ZnONPs onto the clay matrix. The obtained product, known as ZnONPs-Sa, was crushed into fine particles and was then dissolved in an aqueous solution of phenylalanine (Phe) (2 mg/mL) for the functionalization procedure to induce selective interactions with acetaminophen. The functionalization procedure was done by continuously stirring the ZnONPs-Sa/Phe suspension at room temperature for 2 h, followed by filtration, drying, and storing in Eppendorf tubes for future uses. Figure 1 shows the different steps used for the synthesis of the electrode material ZnONPs-Sa/Phe.

### 2.3. Preparation of the Working Electrode and Electrochemical Procedure

The bare glassy carbon electrode (GCE) was cleaned after each measurement with alumina powder, rinsed with distilled water, and finally sonicated with a 1:1 ethanol–water mixture (*v*/*v*), before being modified with a suspension of Sa, Phe, ZnONPs-Sa and ZnONPs-Sa/Phe prepared as follows [18]: 5 mg each of Sa, Phe, ZnONPs-Sa and ZnONPs-Sa/Phe was dispersed in 1 mL of distilled water using ultrasound for 10 min. Five μL of each suspension was applied dropwise to the shiny surface of the bare GCE, then allowed to dry in an oven for 10 min at a temperature of 50 °C. The modified electrodes obtained were labeled GCE/Sa, GCE/Phe, GCE/ZnONPs-Sa, and GCE/ZnONPs-Sa/Phe. These electrodes were then characterized by cyclic voltammetry (CV) and electrochemical impedance spectroscopy (EIS), while differential pulse voltammetry (DPV) was used to quantify the ACOP analyte. In a typical three-electrode electrochemical setup, both bare and modified glassy carbon electrodes (GCE) were used as the working electrode, the reference electrode was an Ag/AgCl electrode (KCl, 3 M), the auxiliary electrode was a platinum wire, and the electrochemical cell contained 10 mL of working solution. CV experiments were recorded for 1 mM of [Fe(CN)_6_]^3−^ and [Ru(NH_3_)_6_]^3+^ (in 100 mM KCl, pH 7.0), and 0.2 mM of ACOP (in 100 mM Britton Robinson Buffer, BRB, pH 5.0), over potential windows of −0.45 to 0.8 V, −0.5 to 0.1 V, and 0.0 to 1.0 V, respectively, at a scan rate of 50 mV/s. DPV experiments were performed within the potential range of 0.1 to 0.8 V: step potential of 15 mV, modulation amplitude of 75 mV, equilibrium duration of 10 s, interval time of 0.5 s, and modulation time of 0.05 s. A Palmsens3 potentiostat was used to perform the EIS measurements at a fixed voltage of 0.01 V over a frequency range of 0.01 Hz to 100 kHz, controlled via PStrace software (version 4.8), while a μ-Autolab type III potentiostat interfaced with GPES software (version 4.9) was used for ACOP electroanalysis. Origin software versions 6.0 and 9.0 was used to analyze data and create graphical representations. A Metrohm 780 pH meter with a glass electrode was used to control the pH during the electrochemical experiments. For the real sample analysis, ACOP was analyzed using the DPV method in two commercial pharmaceutical tablets, with the Africure and doliprane brands, obtained from local pharmacies. In total, 604 mg of Africure and 1088 mg of Doliprane were finely crushed with a mortar to produce a uniform powder, followed by their dissolution in the supporting electrolyte solution (phosphate buffer, 0.1 M, pH 2.0), and the standard addition method was used for quantification using DPV.

### 2.4. Equipment for Physicochemical Characterization

A UV-2450 spectrophotometer was used to record the UV-visible spectra between 200 and 800 nm during the synthesis of nanoparticles embedded onto clay. Fourier transform infrared (FTIR) spectroscopy was performed using a Bruker Vertex 70 spectrometer (Bruker Corp., Billerica, MA, USA) at room temperature to determine the functional groups present on the materials in the region between 4000 and 600 cm^−1^. A Zeiss SIGMA field emission scanning electron microscope (FESEM) and a Philips XL30 (Philips Electron Optics, Eindhoven, The Netherlands) fitted with energy-dispersive X-ray spectroscopy (EDX) were used for the morphology and elemental mapping characterization of materials, respectively, while a Scientific Flash 2000 analyzer (Thermo Fisher Scientific, Milan, Italy) was also used for elemental analysis. X-ray diffraction (XRD) patterns were acquired at room temperature using a D/teX Ultra detector (Rigaku corporation, Tokyo, Japan) with CuKα radiation (λ = 1.5406 Å) for the material crystallinity determination.

## 3. Results

### 3.1. Physicochemical Characterizations of Sa, ZnONPs-Sa, and ZnONPs-Sa/Phe

**Figure 2:**
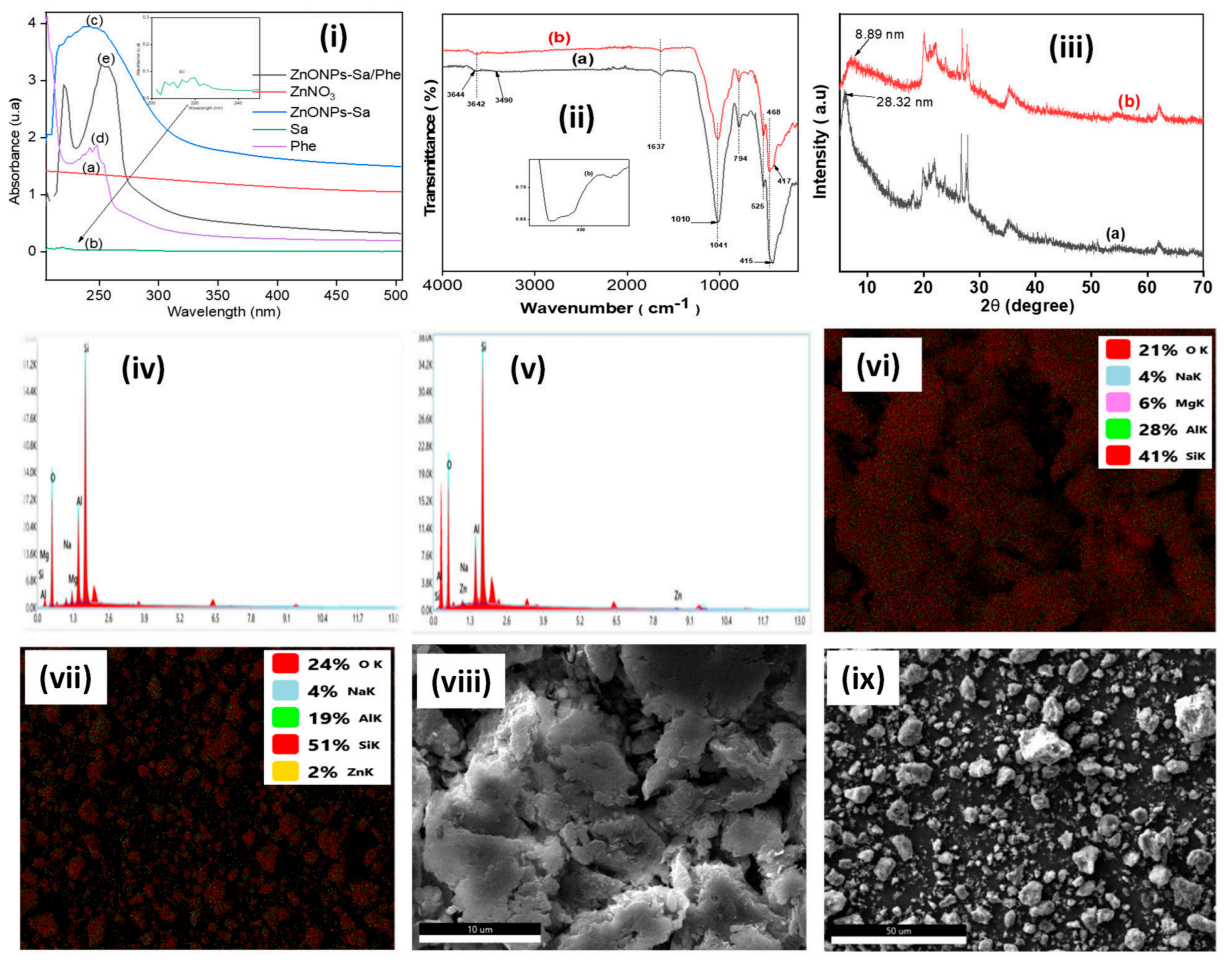
(**i**) UV-vis spectra of (a) Zn(NO_3_)_2_.6H_2_O, (b) Sa, (c) ZnONPs-Sa, (d) Phe, and (e) ZnONPs-Sa/Phe; (**ii**) FTIR spectra of (a) Sa and (b) ZnONPs-Sa; (**iii**) XDR of (a) Sa and (b) ZnONPs-Sa; EDX spectra of (**iv**) Sa and (**v**) ZnONPs-Sa; SEM mapping of (**vi**) Sa and (**vii**) ZnONPs-Sa; FESEM of (**viii**) ZnONPs-Sa and (**ix**) ZnONPs-Sa/Phe. Inset in (**i**-b) shows the zoom spectrum from 200 to 250 nm.

Figure 2i-a shows the UV-Vis spectrum of the zinc nitrate precursor, in which no discernible absorption band is observed, suggesting that there is no notable electronic transition in the scanned wavelength range. On the other hand, an absorption band around 197 to 267 nm can be seen in the montmorillonite clay (Sa) spectrum (Figure 2i-b, inset). This band is mainly attributed to vibrational transitions of surface hydroxyl functionalities (Si-OH) and π → π* electronic transitions linked to organic moieties adsorbed onto the clay surface [27]. After incorporation of ZnO into the sodium montmorillonite matrix, the UV-Vis spectrum of the resulting material shows a notable increase in absorbance in the 250–350 nm region, which is assigned to the formation of ZnONPs in the clay (Figure 2i-c). This increase in absorbance is indicative of the existence of surface-bound zinc hydroxide species or amorphous ZnO phases [28]. The UV-visible spectrum of phenylalanine (Figure 2i-d) exhibits two characteristic absorption bands in the ultraviolet region. A strong absorption band is observed between 200 and 210 nm, which is attributed to the π → π* electronic transition of the aromatic benzene ring. A second, less intense band appears between 255 and 260 nm, also corresponding to π → π* transitions within the aromatic system. No significant absorption is detected beyond 280 nm, indicating the absence of highly conjugated chromophores capable of absorbing in the visible region [29,30]. Interestingly, the spectrum (Figure 2i-e) shows a more intense absorption in this region after functionalization with phenylalanine (ZnONPs-Sa/Phe). This is indicative of electronic perturbations caused by the interaction between Zn^2+^ centers and the functional moieties (-NH_2_ and -COOH) of phenylalanine. Strong interfacial interaction between the clay substrate, zinc oxide nanoparticles, and the amino acid ligand is confirmed by these spectra alterations, which also offer convincing proof of effective nanocomposite formation. Furthermore, the large background signal observed in the spectra may be related to the presence of ZnONPs and organic components in the composite, as well as surface heterogeneity. These factors can contribute to baseline distortion and scattering effects.

Figure 2ii shows the Fourier transform infrared (FTIR) spectra of the ZnONPs-Sa composite and pure montmorillonite (Sa). The stretching vibrations of hydroxyl (-OH) groups, either from physisorbed water molecules or structurally bonded hydroxyls within the clay matrix (Figure 2ii-a), are responsible for the broad absorption band shown in both profiles at about 3640 cm^−1^ [31]. A little shift and decrease in the intensity of this band (occurring at 3642 cm^−1^) in the ZnONPs-Sa spectrum (Figure 2ii-b) is observed, suggesting interactions between hydroxyl functionalities and ZnO nanoparticles that change the local chemical environment [32]. The bending vibrations of molecular water (H-O-H) or potentially aromatic C=C stretching modes are linked to a band at 1637 cm^−1^ that is consistently present in both spectra [33]. Additionally, the ZnONPs-Sa composite’s attenuation of the Si-O stretching vibration at 1010 cm^−1^ in comparison to the unmodified clay indicates the development of structural interactions between ZnO and the silicate framework, especially through the Si-O-Si bridge. Layered alumino-silicate minerals are characterized by discrete bands that correspond to Si-O and Al-O-Si vibrational modes in the lower wavenumber range (790–400 cm^−1^) [34]. The stretching vibrations of Zn-O bonds are responsible for the emergence of unique absorption bands at 524 and 468 cm^−1^ in the ZnONPs-Sa spectra, which provides conclusive spectroscopic evidence for the successful synthesis of ZnO nanoparticles on the clay surface [35]. X-ray diffraction (XRD) analysis of sodium montmorillonite (Figure 2iii-a) reveals a characteristic basal (001) reflection typical of smectite clays, confirming the lamellar structure of the material. The relatively sharp diffraction peaks indicate a well-developed structural organization, with an average crystallite size estimated at 28.32 nm using the Scherrer equation. Following ZnO incorporation (sample Figure 2iii-b), the XRD pattern exhibits pronounced modifications. The shift in the basal (001) reflection toward lower 2θ angles indicates an increase in interlayer spacing, providing clear evidence of ZnO intercalation and/or encapsulation within the montmorillonite layers. A significant broadening of the main diffraction peaks is observed for sample (b) in Figure 2iii, reflecting a substantial reduction in crystallite size. The average ZnO crystallite size is estimated to be 8.89 nm, highlighting the confinement effect imposed by the montmorillonite matrix. This confinement restricts ZnO crystal growth and promotes controlled nucleation, resulting in finely dispersed ZnO nanocrystallites within the clay structure [36].

The elemental analysis results (Table 1) show that silicon (39.6%), aluminum (13.6%), and oxygen (43.0%) are predominant in pristine sodium clay, Sa(Na). This is consistent with the typical stoichiometry of alumino-silicate minerals like smectite or sodium montmorillonite [37]. The small amounts of exchangeable cations in the interlayer regions are reflected in the quantities of sodium (1.7%) and magnesium (2.1%). The ZnONPs-Sa(Na) composite exhibits a significant increase in oxygen content (48.3%) and the appearance of zinc (1.2%) upon integration of ZnO nanoparticles, confirming the successful integration of ZnO inside the clay framework. At the reactive sites of clay lattice, this incorporation most likely occurs by surface adsorption or ion-exchange processes [38]. A displacement process is further supported by the simultaneous decrease in sodium (0.2%) and total depletion of magnesium (0.0%), indicating that Zn^2+^ ions partially replace these native cations during the synthesis.

These results are confirmed by the EDX analysis in which the spectrum of the raw clay (Figure 2iv) shows discrete peaks corresponding to silicon (Si), aluminum (Al), sodium (Na), magnesium (Mg), and oxygen (O). On the other hand, a noticeable zinc (Zn) peak appears in the ZnONPs-Sa spectrum (Figure 2v) at about 8.6 keV, confirming the integration of ZnO nanoparticles into the clay framework. Additionally, elemental mapping images (Figure 2vi,vii) show a uniform zinc distribution throughout the composite material, suggesting effective and consistent nanoparticle dispersion across the surface of the clay matrix.

Field emission scanning electron microscopy (FESEM) was used to examine the surface morphology of the prepared nanomaterials. Significant particle clustering is seen in the FESEM image of the ZnONPs-Sa (Figure 2viii), which shows tightly packed and asymmetrical agglomerates. Strong interfacial contacts between the ZnO nanoparticles and the montmorillonite clay support (Sa) are reflected in this tight architecture, which probably contributes to the hybrid material’s increased mechanical robustness. However, this type of aggregation, which is often seen in unaltered nanostructured systems, can result in a decrease in the effective surface area, which may limit the material’s capacity for catalysis and adsorption [39]. On the other hand, the ZnONPs-Sa/Phe nanocomposite’s FESEM image (Figure 2ix) shows a considerable change in surface morphology, with a more uniform particle distribution and much less agglomeration [40]. Phenylalanine’s surface active function, which successfully reduces nanoparticle clumping by introducing steric hindrance and intermolecular interactions, is responsible for this morphological refinement [41]. Increased accessible surface area from the improved dispersion promotes better interfacial interactions and could lead to better photocatalytic and electrochemical performance [42]. These findings highlight the usefulness of phenylalanine as a stabilizing and dispersing agent, providing a tactical edge in adjusting the surface structure of ZnO-based hybrid materials.

### 3.2. Electrochemical Characterization of Sa, ZnONPs-Sa, and ZnONPs-Sa/Phe

**Figure 3:**
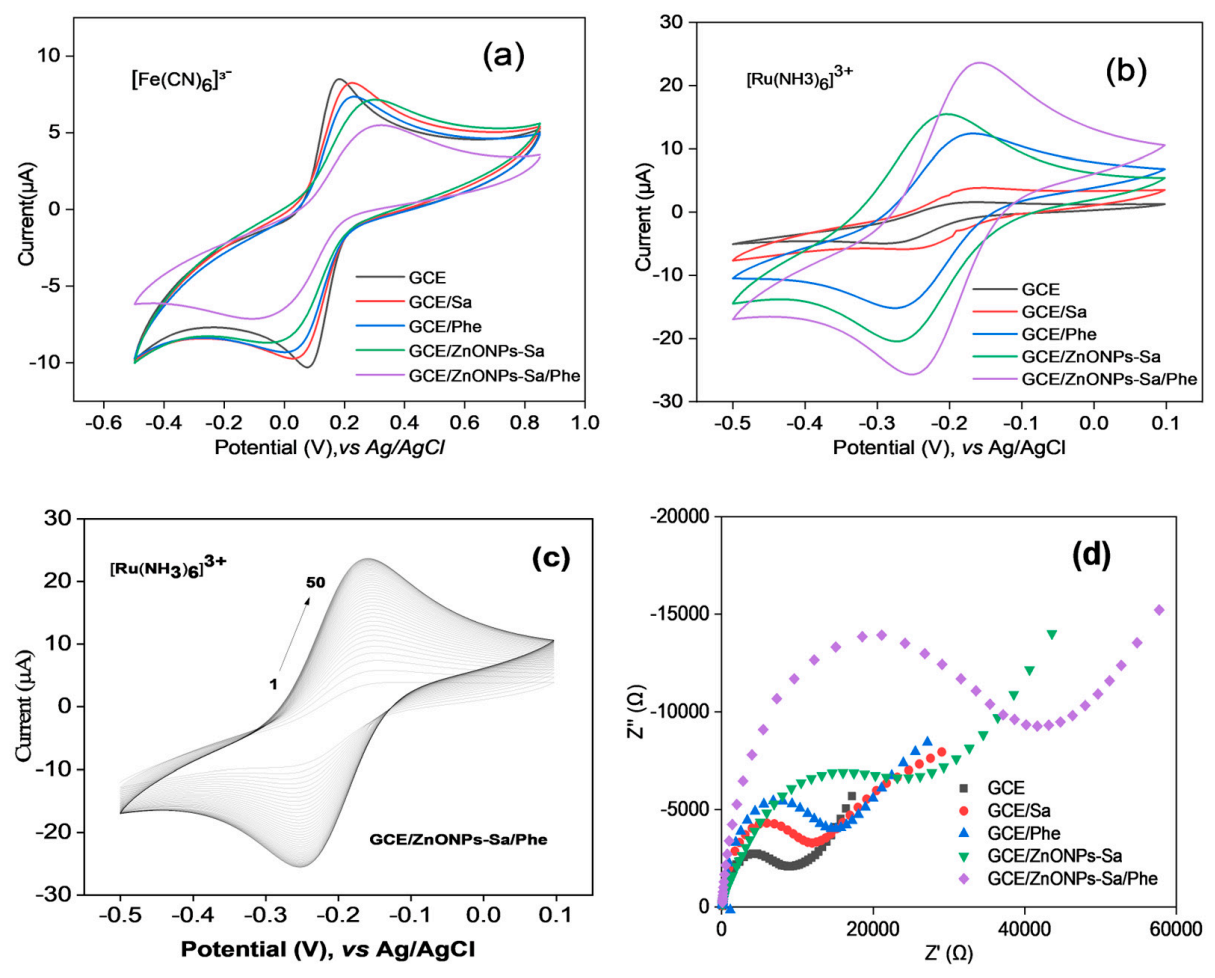
Cyclic voltammograms recorded in a 0.1 M KCl solution containing (**a**) 10^−3^ M [Fe(CN)_6_]^3−^ and (**b**) 10^−3^ M [Ru(NH_3_)_6_]^3+^ at pH 5 on different electrodes: bare GCE, GCE/Sa, GCE/Phe, GCE/ZnONPs-Sa, and GCE/ZnO-Sa/Phe, with a scan rate of 50 mV/s. (**c**) Multicyclic voltammograms (50 scans) recorded in a 0.1 M KCl solution containing 10^−3^ M [Ru(NH_3_)_6_]^3+^ at pH 5 on GCE/ZnO-Sa/Phe. (**d**) Nyquist diagrams obtained in 10^−3^ M [Fe(CN)_6_]^3−/4−^ on the same electrodes.

The redox probes [Fe(CN)_6_]^3−^ and [Ru(NH_3_)_6_]^3+^ were used for the characterization of bare GCE, GCE/Sa, GCE/Phe, GCE/ZnONPs-Sa, and GCE/ZnO-Sa/Phe using cyclic voltammetry (CV) and electrochemical impedance spectroscopy (EIS). These probes were chosen for their opposite charges and well-defined electrochemical responses, enabling the investigation of surface charge and electron transfer processes. The cyclic voltammograms obtained in a neutral medium with the anionic redox probe [Fe(CN)_6_]^3−^ are shown in Figure 3a. The bare GCE (Ipa = 7.5 µA) showed the most intense redox peak currents, and the smallest current obtained with GCE/ZnONPs-Sa/Phe (Ipa = 6 µA) indicates a strong electrostatic repulsion between the negatively charged ferri/ferrocyanide species and the film. This could help to conclude that the hybrid material deposited at the surface of bare GCE has a globally negative charge surface due to the presence of a clay material known to have an average negative charge surface [24,43]. The existence of observable redox signals indicates that the modified surface still exhibits a heterogeneous distribution of surface charges. However, as expected for the positive redox probe [Ru(NH_3_)_6_]^3+^ (Figure 3b), a strong redox peak was observed with GCE/ZnONPs-Sa/Phe (Ipa = 25 µA) compared to bare GCE (Ipa = 5 µA), which is assigned to the strong electrostatic attraction between the negatively charged functional groups of the hybrid film and the positively charged ruthenium complex. Additionally, this result could be explained by hydrogen bonding and π-π interactions arising from the organic functionalities introduced by phenylalanine. These functionalities improve analyte adsorption and facilitate more effective electron transfer [32,42]. Furthermore, as shown in Figure 3c, the porous shape of the ZnONPs-Sa/Phe composite improves the slow buildup of [Ru(NH_3_)_6_]^3+^ at the electrode interface. The high affinity and significant adsorption capacity of the modified electrode material toward of the positive redox probe is confirmed by the gradual increase in the peak current intensity with the scan number from the 1st to the 40th cycle, demonstrating a strong accumulation behavior of GCE/ZnONPs-Sa/Phe.

The charge transfer resistance (Rct) of the different modified electrodes was then evaluated using electrochemical impedance spectroscopy (EIS) analysis. As illustrated in Figure 3d, the Nyquist plots indicated that in the presence of [Fe(CN)_6_]^3−^, the GCE/ZnONPs-Sa/Phe exhibited the highest Rct value (50 kΩ) compared to the other electrodes, which corroborates the results obtained from CV analysis (Figure 3a). As expected, the bare GCE showed the lowest Rct value (10 kΩ), suggesting more effective charge transfer with the anionic species. These findings highlight the electrochemical selectivity of the ZnONPs-Sa/Phe composite, which limits the electron transfer for anionic species due to electrostatic repulsion and decreased surface accessibility, and improves the response towards cationic analytes via attractive interactions [35,44].

### 3.3. Electrochemical Determination of ACOP

#### 3.3.1. Electrochemical Behavior and Kinetic Study of ACOP

**Figure 4:**
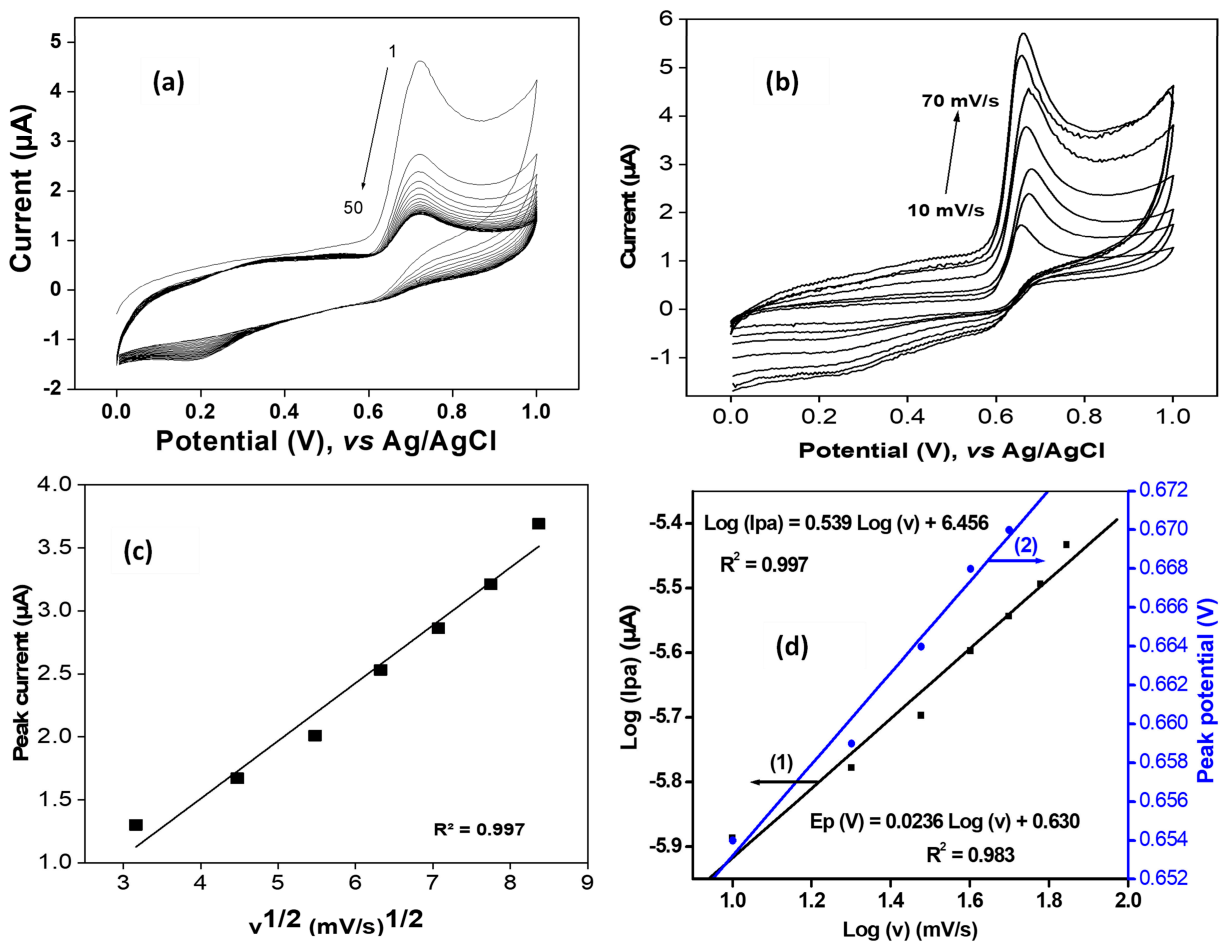
(**a**) Multicyclic voltammograms of 10^−4^ M ACOP in 0.1 M PB, pH = 2.0 at GCE/ZnONPs-Sa/Phe, scan rate: 50 mV/s. (**b**) Cyclic voltammograms obtained in 0.1 M KCl (pH 2.0) containing 10^−4^ M ACOP on GCE/ZnONPs-Sa/Phe at different scan rates from 10 to 70 mV/s. (**c**) Peak current versus v^1/2^, (**d**) (1) Log Ipa = f (Log v); (2) Ep = f (Log v).

The redox behavior of ACOP was examined by recording 50 cyclic voltammograms using the electrode modified with ZnONPs-Sa/Phe composite, as seen in Figure 4a. The figure shows that ACOP undergoes a quasi-reversible electrochemical process, with a distinct anodic peak current of 2.37 µA at a potential of 0.72 V and a less noticeable cathodic peak current of 0.04 µA at a potential of 0.20 V (appearing after seven cycles). The restriction in the ability of the oxidized species to be regenerated at the electrode surface is the cause of the poor reduction signal, as previously reported [45]. The peak current oxidation gradually decreases with the increase in the scan number, suggesting that the electrode surface was passivated from the second scan. This effect could be assigned to the ACOP oxidation products, which block the pores of ZnONPs-Sa/Phe film responsible for the oxidation and prevents access to catalytic sites [46]. The electrode surface was refreshed before each measurement to prevent the passivation effect affecting the results.

An electrochemical process governed by both charge transfer kinetics and mass transport phenomena is shown in Figure 4b, where the anodic peak current rises proportionally to the scan rate while the peak potential shifts toward more positive values. Figure 4c shows a slope of 4.584 obtained from the linearity of the plot of the anodic peak current (Ipa) against the root square of the scan rate (v^1^/^2^) according to the Randles–Ševčík equation (Ip = 0.4463 n.F.A.C($\frac{nF.v.D}{RT}{)}^{1/2}$) [47]. This linearity (R^2^ = 0.997, close to 1) confirms that there is a good correlation between the peak current and the square root of the scan rate, which is a feature of a diffusion-controlled process. Furthermore, Figure 4(d1) shows the plot of anodic peak current (Ipa) vs. log (v), yielding a slope of 0.538, which is close to the anticipated theoretical value of 0.5 for a solely diffusion-controlled process [14,48,49]. By plotting the peak potential (Ep) vs. log (v) given by Figure 4(d2) (only for the scan rate from 10 to 50 mV/s, as the potential was not changing at a high scan rate of 60 mV/s and 70 mV/s), the value of the slope obtained from the Laviron equation [49,50,51] (Equation (1)) is 24 mV/decade.

(1) $$E_{P}=E^{0}+\left(\frac{2.303RT}{\alpha nF}\right)log\left(\frac{RTK^{0}}{\alpha nF}\right)+\left(\frac{2.303RT}{\alpha nF}\right)log(v)$$

where α represents the transfer coefficient; n, the number of electrons exchanged; v, the scan rate; K, the heterogeneous rate constant; T, the absolute temperature; R, the gas constant; and E, the standard potential obtained by extrapolation on the potential axis when v = 0 mV/s. From Equation (1), we obtained n = 4.10 ≈ 4, assuming α to be 0.55 [50], which suggests the use of four electrons.

The Randles–Ševčík equation was used to compute the diffusion coefficient (D) in view of verifying the diffusion-controlled mechanism of ACOP on the ZnONPs-Sa/Phe modified electrode using the cyclic voltammetry data (Figure 4b). The formula used was Ip = 268,600 C [52], taking into account a two-electron transfer (n = 2), an analyte concentration of 10^−4^ mol/L, an electroactive surface area (A) of 0.071 cm^2^, a scan rate (v) of 50 mV/s, a temperature of 25 °C, and an oxidation peak current (Ipa) of 2.3 µA. This led to determining the diffusion coefficient, D = 3.66 × 10^−6^ cm^2^/s. This value confirms that the electrochemical oxidation of ACOP on the ZnONPs-Sa/Phe modified electrode is diffusion-controlled, as described in the literature for comparable systems [48].

#### 3.3.2. Influence of the Electrode Composition, the Modifier Loading, and the Detection Medium

**Figure 5:**
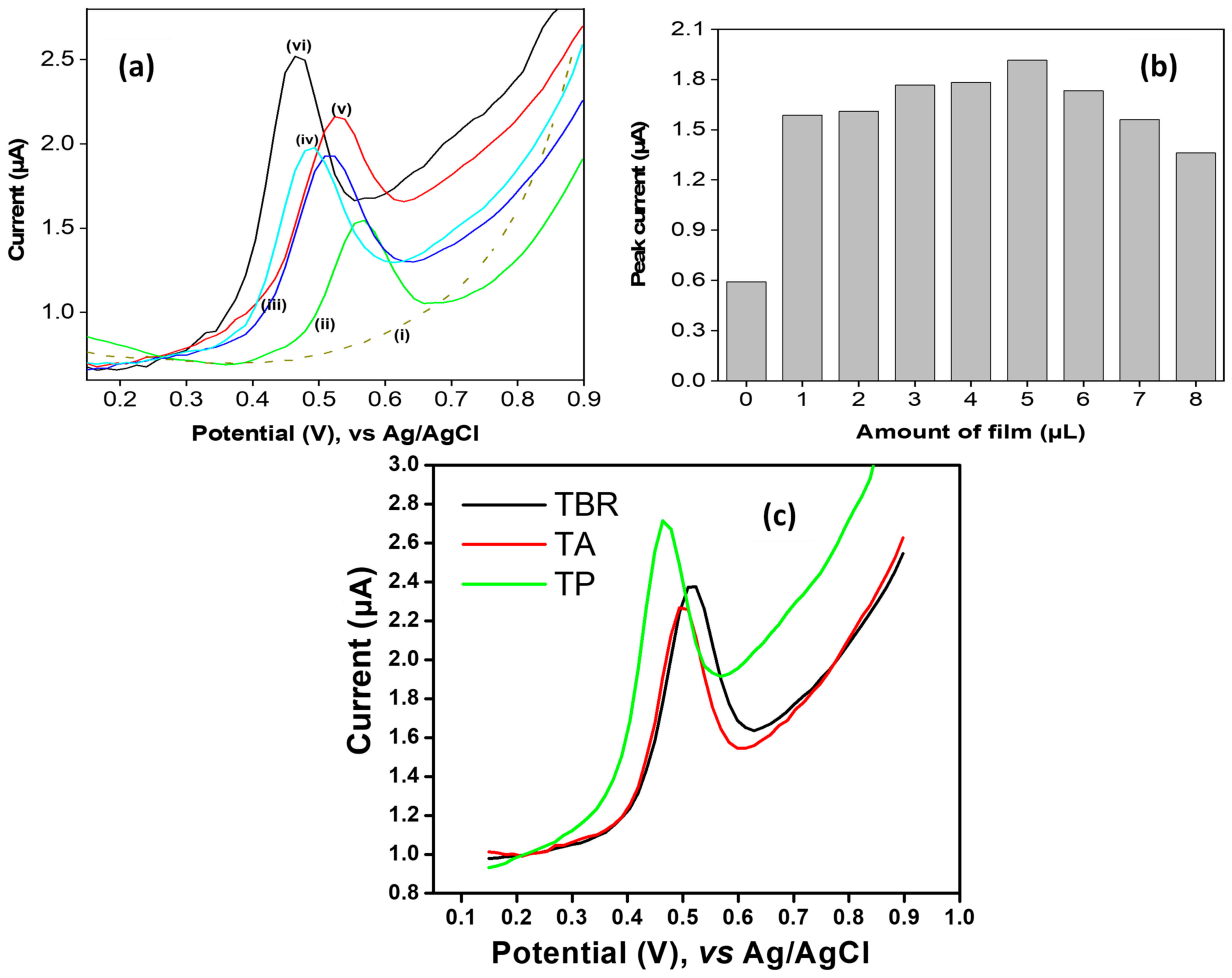
(**a**) DPV response of (ii) bare GCE, (iii) GCE/ZnONPs-Sa, (iv) GCE/Phe, (v) GCE/Sa, and (vi) GCE/ZnONPs-Sa/Phe recorded in 0.1 M PB solution (pH = 2.0) containing 20 µM ACOP with (i) the blank. (**b**) DPV peak current as a function of the amount of ZnONPs-Sa/Phe deposited on the GCE, recorded in 0.1 M PB solution (pH = 2.0) containing 20 µM ACOP. (**c**) Influence of different media (0.1 M PB, AB, BRB solutions at pH = 5.0) on the electrochemical response of ACOP on GCE/ZnONPs-Sa/Phe. The DPV response in the black dashed line corresponds to the blank. Experiments were performed in triplicate.

Differential pulse voltammetry (DPV) was used to analyze the influence of the electrode modification, as shown in Figure 5a. The electrode modified with the GCE/ZnONPs-Sa/Phe composite produced the highest current intensity (2 µA), which is approximately twofold enhance compared to the bare electrode (1 µA). A change in potential from 0.566 V (bare GCE) to 0.466 V (GCE/ZnONPs-Sa/Phe) can also be noticed, suggesting that the improved surface enhanced electron transport (good electrocatalytic ability). Furthermore, acetaminophen is primarily found in its cationic form at pH 2.0, which promotes electrostatic interaction with the negatively charged surface of the composite originate from the clay layers and the oxygenated sites of ZnO. While the Sa clay matrix offers a porous structure that enhances analyte dispersion, ZnO nanoparticles offer excellent electrical conductivity and promote charge transfer.

Figure 5b shows the results of the examination of the film volume deposited on the surface of the glassy carbon electrode (GCE) modified with the ZnONPs-Sa/Phe composite. As the film volume increased up to 5 μL, a progressive rise in peak current was seen, suggesting a notable increase in the density of electroactive sites accessible for ACOP identification and oxidation. This improvement is explained by the homogenous dispersion of ZnO nanoparticles, which promotes electron transport, and the expansion of the electrochemically active surface area, which is supported by the mesoporous structure of the clay matrix [32,35]. On the other hand, a significant drop in peak current is seen when the film volume is more than 5 μL, which is explained by the creation of an overly thick film, functioning as a diffusion barrier and restricting analyte access to the electrode active sites. This over-thickness delays the diffusion of electroactive species and lowers the film overall electronic conductivity, which is a characteristic frequently observed in multilayer-modified systems [14,24]. Consequently, an ideal volume of 5 μL was chosen to guarantee a balance between electrochemical performance, mechanical stability of the film, and catalytic site availability. These results demonstrate how crucial it is to optimize deposition conditions when designing a nanocomposite-based electrochemical sensor to maximize sensitivity and reliability.

Three buffers, namely Britton–Robinson buffer (BRB), phosphate buffer (PB), and acetate buffer (AB), all adjusted to pH 5, were assessed using differential pulse voltammetry (DPV) to identify the best medium for ACOP detection. The DPV responses using GCE/ZnONPs-Sa/Phe are shown in Figure 5c. Compared to BRB (1.374 µA) and AB (1.263 µA), the phosphate buffer (PB) exhibited the greatest oxidation peak current (1.703 µA), suggesting that PB offers the best conditions for ACOP electrooxidation, and also has good chemical stability and reproducibility.

#### 3.3.3. Influence of pH and Calibration Curve

**Scheme 1:**
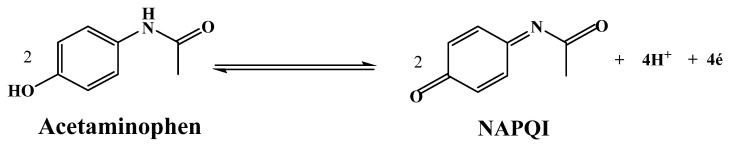
Mechanism of the electrochemical oxidation of acetaminophen.

**Figure 6:**
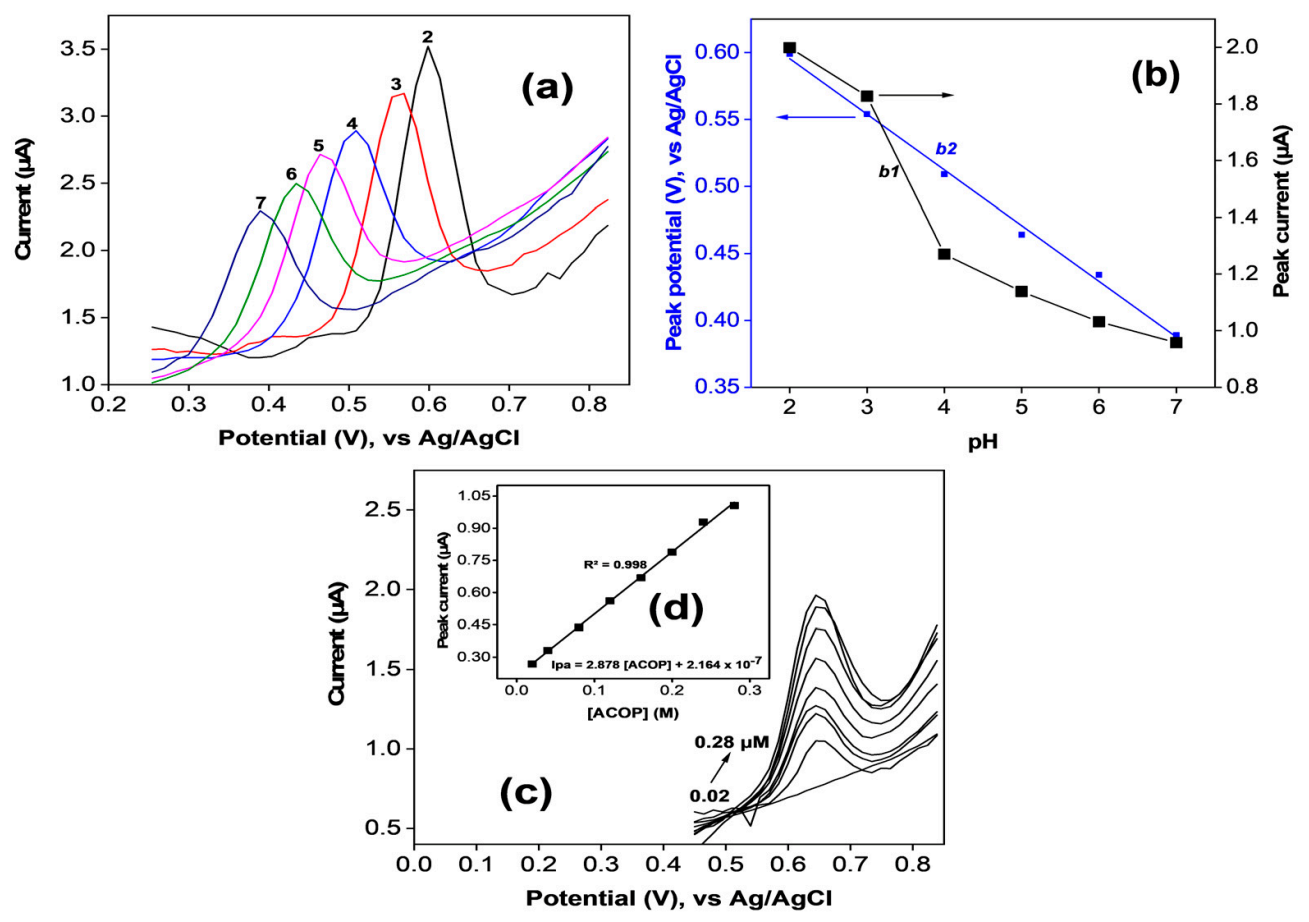
(**a**) Effect of pH of the supporting electrolyte (from 2 to 7) on the electrochemical response of 20 µM ACOP (100 mM BRB) recorded on GCE/ZnONPs-Sa/Phe. (**b**) Plot of: (b1) Peak current as a function of pH and (b2) Peak potential as a function of pH. (**c**) DPV response recorded on GCE/ZnONPs-Sa/Phe in 100 mM PB solution (pH 2.00) containing ACOP at different concentrations from 0.02 µM to 0.28 µM. (**d**) The corresponding calibration graph.

The differential pulse voltammograms of ACOP (20 µM), recorded in phosphate buffer (PB, 100 mM) from a pH ranging from 2.0 to 7.0 using GCE/ZnONPs-Sa/Phe, are shown in Figure 6a. When pH rises, a distinct change toward the negative potentials of the oxidation peak potential (Ep) is observed, suggesting that protons are directly involved in the redox process. According to the Nernstian slope (≈−0.059 V/pH), the linear regression in Figure 6(b2) follows the equation Ep = −0.042 pH + 0.679 (R^2^ = 0.996), which is compatible with a coupled proton–electron transfer involving two electrons and two `protons [14,53]. Knowing that the Laviron equation shows a four-electron exchange, two molecules of ACOP may have participated in the electrochemical oxidation process. The sensor maintains good sensitivity at pH 2.0, which was chosen as ideal for further investigations (Figure 6(b1)). The proton-rich environment at this pH promotes electron transport and stabilizes the quinonoid product (NAPQI) by facilitating the oxidation of the phenolic (-OH) group of ACOP. In the synergistic properties of the ZnONPs-Sa/Phe composite, ZnONPs contribute to electron conductivity, the clay matrix (Sa) provides a porous network for analyte diffusion, and phenylalanine introduces functional groups (-NH_2_, -COOH) that promote hydrogen bonding and π-π interactions with the ACOP aromatic ring, and are further responsible for the enhanced response at low pH [18]. Strong adsorption and effective charge transfer are promoted by these phenomena, and the overall electrochemical reaction can be expressed by Scheme 1 as follows [54].

A linear electrochemical response in the range of 0.02–0.28 µM was used to evaluate the limit of detection (LOD) of the developed electrochemical sensor. Figure 6c shows the voltammograms in which the peak current increases with the concentration. The plot of peak current as a function of concentration (Figure 6d) leads to a linear regression Ipa = 2.878 [ACOP] + 2.164 × 10^−7^, with LOD = 8.54 nM, and a limit of quantification (LOQ) = 28 nM, calculated using the respective formula LOD = 3 Sb/m and LOQ = 10 Sb/m, where Sb is the standard deviation of the blank signal (Sb = 8.19229 × 10^−9^) and m is the slope of the calibration curve (m = 2.878) [55]. These results demonstrate the great sensitivity of the sensor, allowing for trace-level acetaminophen detection in comparison to other work reported in Table 2.

A critical comparison of Table 2 shows that, although the GCE/ZnONPs-Sa/Phe sensor does not exhibit the lowest LOD among the reported systems, it still demonstrates competitive analytical performance (0.00854 µM), comparable to several previously reported sensors, such as AuNP/NCNO (0.009 µM) and [Co(5,5-dmbipy)_2_(NCS)_2_]-SPE (0.005 µM). While some sensors achieve lower LODs, these often rely on more complex fabrication procedures, expensive materials (e.g., noble metals), or less practical operating conditions. In contrast, the present sensor offers several notable advantages. The use of ZnO-based nanomaterials provides a cost-effective, easily synthesized, and environmentally benign platform. The electrode modification strategy is relatively simple compared to multi-component or highly engineered nanocomposites reported in the literature, which enhances reproducibility and scalability. Additionally, the sensor demonstrates reliable performance in a practical medium (TP), supporting its applicability for real sample analysis.

#### 3.3.4. Interference Study, Reproducibility, Repeatability and Stability of GCE/ZnONPs-Sa/Phe

**Figure 7:**
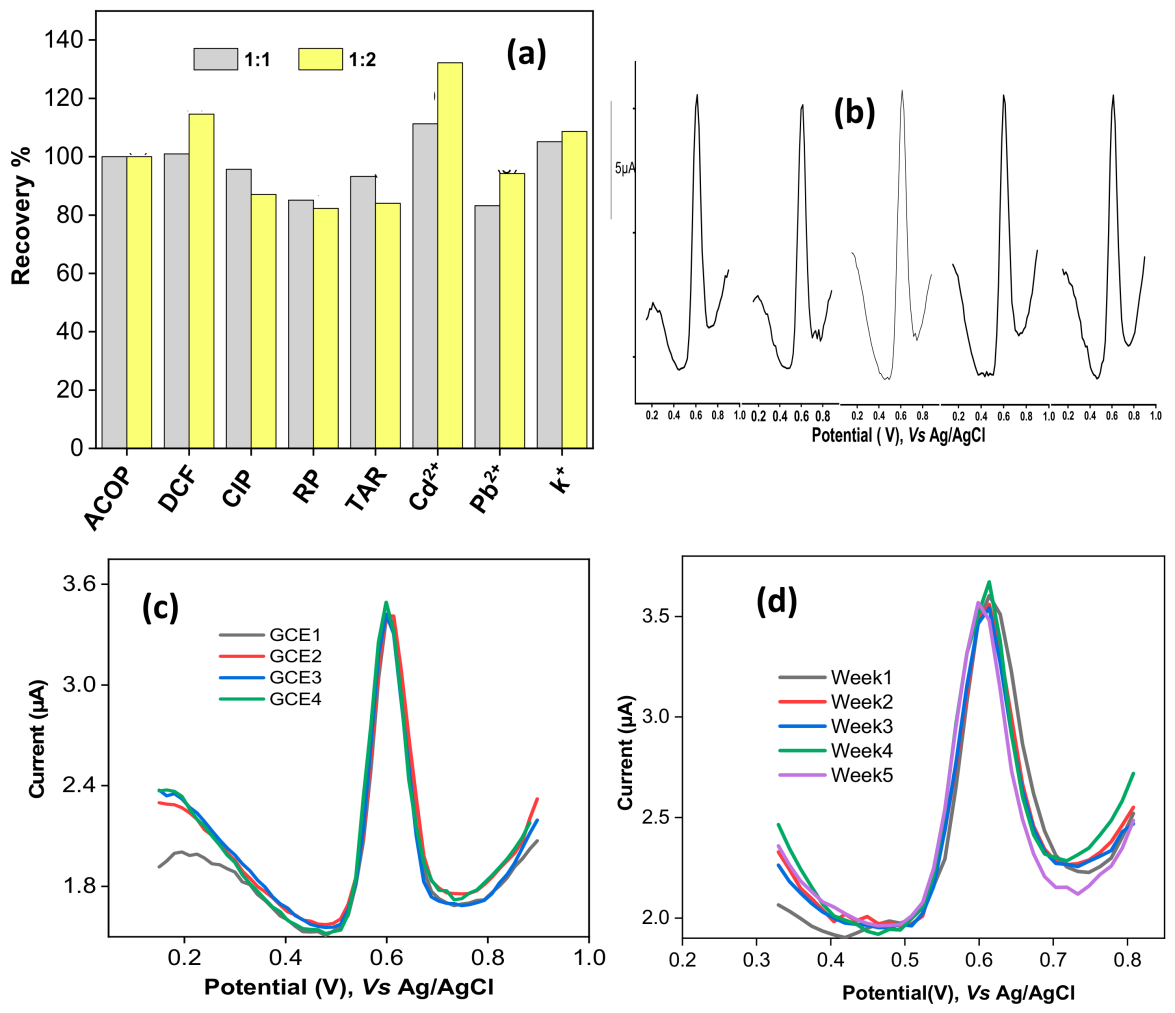
(**a**) Effect of potentially interfering species on the peak currents of ACOP recorded in PB solution (pH = 2) on GCE/ZnONPs-Sa/Phe. Differential pulse voltammograms of 20 µM acetaminophen recorded on a GCE /ZnONPs-Sa/Phe at pH 2.0, showing the (**b**) reproducibility, (**c**) repeatability, and (**d**) stability of the signal.

The selectivity of the developed sensor was assessed by recording the oxidation peak of ACOP in the presence of several possible interfering compounds, such as diclofenac (DCF), tartrazine (TAR), ponceau 4R (RP), and ciprofloxacin (CIP), as well as metallic ions (K^+^, Pb^2+^, Cd^2+^), which are frequently present in real samples. Although they might not accurately reflect all potential electroactive species found in real matrices, the chosen interferents offer an initial assessment of the sensor selectivity. When these interfering species were added at equimolar or twofold concentrations, the peak current response for 20 µM ACOP changed from 85% to 130%, suggesting that some species do not affect the recovery, while others affect the selectivity of the sensor by decreasing or increasing its detection efficiency, as demonstrated in Figure 7a (see the corresponding voltammograms in supporting information, Figure S1). For instance, diclofenac clearly interfered by increasing the recovery to 110% and Cd^2+^ to 130%. This is probably because ACOP and DCF have overlapping oxidation potentials and can both undergo electrochemical oxidation, and because Cd^2+^ may produce an ionic effect that facilitates the detection of ACOP at the surface of the electrode. Conversely, a decrease in the ACOP signal in the presence of CIP, TAR, and RP could be caused by electrode surface occupation or competitive oxidation processes, impeding its electrochemical response. To improve the selectivity of the developed sensor, other materials with a good electro-catalytic effect could be used, such as multiwalled carbon nanotubes and reduced graphene oxide. Other potential ways to reduce such interferences include optimizing the applied potential window, altering the electrode surface to improve selectivity, and using chemometric techniques for signal resolution. To thoroughly assess the selectivity of the suggested sensor, more research involving actual metabolites and intricate pharmaceutical excipients is needed. Although K^+^, Pb^2+^ and Cd^2+^ were used to assess preliminary selectivity, these ions do not accurately reflect the ionic composition of human serum. Future research will involve a more thorough investigation using additional ions and biomolecules to evaluate the sensor’s functionality in physiological settings. These findings demonstrate the significance of taking into account molecular structure and redox potential overlaps when analyzing complex mixtures. Therefore, prior to using the developed sensor in real environmental samples, we should make sure that the milieu does not contain the compounds that interfere with the target compound. Good inter-electrode reproducibility is confirmed by the ensuing a relative standard deviation (RSD%) of 4.07%, underscoring the reliability of the fabricated method. Five GCE/ZnONPs-Sa/Phe electrodes were independently produced using the same technique, and the reproducibility of the sensor was evaluated for the detection of 20 µM ACOP (Figure 7b). By doing four consecutive DPV measurements on the same modified electrode under the same circumstances, the repeatability of the sensor was evaluated. For the elimination of any remaining ACOP without removing the film, the electrode was washed with phosphate buffer (100 mM, pH 2.00) between each measurement (Figure 7c). Excellent repeatability and signal stability during subsequent studies were demonstrated by the RSD of 1.64%. After being properly cleaned and stored at room temperature between usage, a modified electrode was tested once a week for five weeks to ensure long-term stability. In 100 mM phosphate buffer at pH 2.0, a DPV signal of 20 µM ACOP was obtained (Figure 7d). The appropriateness of the sensor for routine monitoring is confirmed by the computed RSD of 7.15% throughout this period, which shows that it maintains acceptable stability for up to four weeks.

#### 3.3.5. Real Sample Analysis Using the GCE/ZnONPs-(Sa)/Phe Sensor

**Figure 8:**
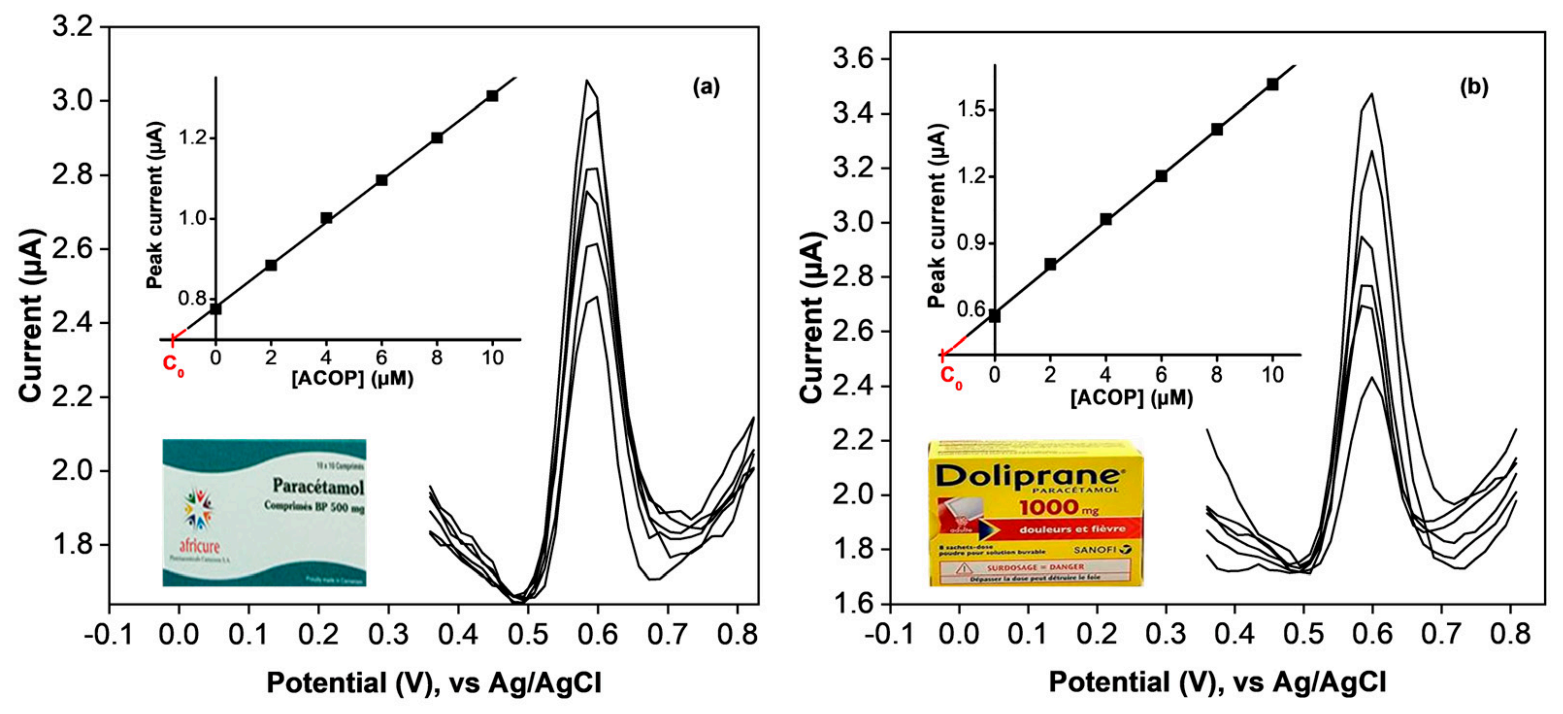
Differential pulse voltammograms recorded for the commercial (**a**) Africure and (**b**) Doliprane capsule after addition of known amounts of ACOP and corresponding calibration curve inset. The insets indicate the corresponding calibration curves.

For quality control reasons, the modified electrochemical sensor GCE/ZnONPs-(Sa)/Phe was effectively used to quantify ACOP in commercial pharmaceutical tablets. To evaluate the analytical performance of the sensor in actual matrices, two distinct brands sold in pharmacies, such as Africure and Doliprane, were selected. The concentration range covered by the usual addition procedure was 2 µM to 10 µM. As the concentration of ACOP increased, the corresponding voltammograms (Figure 8a,b) showed a proportional increase in peak current, resulting in linear calibration plots (insets of Figure 8a,b). The following linear regression equations were found: Africure: Ipa (µA) = 0.78067 [ACOP] + 52,645.71; Doliprane: Ipa (µA) = 0.57673 [ACOP] + 104,544.29, where C_add_ is the additional amount and [ACOP] = C_0_ + C_add_ is the overall concentration of acetaminophen (mol/L). Recovery rates of 100.84% for Africure and 97.18% for Doliprane were found in the experimental data reported in Table 3, demonstrating the excellent precision and reliability of the suggested approach. These results validate the potential of the GCE/ZnONPS-Sa/Phe sensor as a sensitive and effective platform for ACOP detection in actual pharmaceutical formulations.

## 4. Conclusions

In conclusion, a novel electrochemical sensor based on a glassy carbon electrode (GCE) modified with zinc oxide nanoparticles (ZnONPs) embedded in phenylalanine-coated montmorillonite clay (Sa) was successfully developed for sensitive and selective detection of acetaminophen (ACOP). The synthesized material was characterized by FTIR, UV-Vis, XRD, FESEM/EDX mapping, and electrochemical techniques. The results revealed the successful formation of ZnO nanoparticles, their homogeneous dispersion within the clay matrix, and the effective surface functionalization by Phe. The synergistic effect between ZnONPs, the clay support, and phenylalanine significantly contributed to improving the sensitivity and selectivity of the sensor. According to kinetic testing and diffusion coefficient calculations (D = 3.66 × 10^−6^ cm^2^/s), the modified electrode demonstrated good electroanalytical performance in acidic phosphate buffer (pH 2.0), with a mostly diffusion-controlled process and slight surface adsorption. The sensor demonstrated a broad linear dynamic range (0.02–0.28 µM), a low limit of detection (LOD = 0.00854 µM), exceptional repeatability, acceptable reproducibility, and remarkable stability over a five-week period. Additionally, the practical application of the sensor for real sample analysis was validated with accurate recovery rates (97–101%) in commercial pharmaceutical tablets (Africure and Doliprane), demonstrated by the synergy between ZnO nanoparticles, the clay matrix (Sa), and functional amino acid (Phe), which greatly improved the electrocatalytic properties. Moreover, the lack of applicability of the sensor in more complex media with related compounds such as real metabolites and/or complex pharmaceutical excipients limits the applicability of the developed sensor in complex media for clinical diagnosis. Also, the lack of comparison of this validation method with other standard methods, such as HPLC, constitutes a weakness of this work, which could be improved upon in future work. Therefore, the miniaturization of this GCE/ZnONPs-Sa/Phe system could be considered as a potent and economical platform for routine quality control and trace-level detection of ACOP in complex media.

## Figures and Tables

**Table 1:** Elemental analysis of Sa(Na) and ZnONPs-Sa.

Materials	O%	Na%	Mg%	Al%	Si%	Zn%
Sa(Na)	43.0	1.7	2.1	13.6	39.6	/
ZnONPs-Sa	48.3	0.2	0.0	10.2	40.1	1.2

**Table 2:** The linear concentration range and LOD value of the suggested method are compared to a few sensors found in the literature.

Electrode/Modifier	Method	pH	Medium	Linear Range (µM)	LOD (µM)	Reference
GCE/Sm_2_O_3_@ZrO_2_/NTC	VOC	7.0	TP	0.0037–2.2	0.00034	[56]
Yb_2_O_3_-SPEs	DPV	9.0	TP	0.25–654	0.055	[57]
GCE/mpg-CN/BP-Au	DPV	7.4	TP	0.3–120	0.042	[13]
GNM/CPE	DPV	7.4	TP	0.05–270	0.0146	[58]
GCE/gCN-AgPVP	VOC	6.1	TP	0.2–100	0.079	[59]
EPC-PaT	DPV	7.4	TP	6.6–500	3.9	[60]
ZnONPs/SBA-15/GCE	DPV	5.0	BRB	4–32	0.11	[14]
3AT-GCE	DPV	7.0	TP	0.108–89.5	0.043	[61]
4HQ3CA/GC	DPV	1.0	BRB	0.0025–141	0.00059	[53]
35DT-GCE	DPV	1.5	HClO_4_^−^	0.3–55	0.1	[62]
[Co(5,5′-dmbipy)^2^(NCS)_2_]-SPE	DPV	7.0	TP	0.09–325	0.005	[63]
Protonated g-C_3_N_4_/CTs-GCE	DPV	7.0	TP	1–50	0.49	[64]
AuNP/NCNO	DPV	7.0	TP	0.025–35	0.009	[65]
GCE/ZnONPs-Sa/Phe	DPV	2.0	TP	0.02–0.28	0.00854	This work

GCE/gCN-AgPVP: Graphitic Carbon Nitride–Polyvinylpyrrolidone Nanocomposite-Modified Glassy Carbon Electrode with Silver; GCE/Sm_2_O_3_@ZrO_2_/NTC: Carbon Nanotube Composite with Samarium Oxide and Zirconium Oxide Nanoparticles on a Glassy Carbon Electrode; GCE/mpg-CN/BP-Au: Ternary Nanocomposite of Mesoporous Graphitic Carbon Nitride, Black Phosphorus, and Gold Nanoparticles on a Glassy Carbon Electrode; EPC-PaT: Carbon Paste Electrode Modified with Coffee Parchment; GNM/CPE: Carbon Paste Electrode Modified with Gold Nanoparticles; Yb_2_O_3_-SPEs: Screen-Printed Electrodes Modified with Nanostructured Ytterbium Oxide Nanoplates: 3AT-GCE: 3-Amino-4H-1,2,4-Triazole-Coated Glassy Carbon Electrode; 35DT-GC: 3,5-Diamino-1,2,4-Triazole on Glassy Carbon Electrode; ZnONPs/SBA-15/GCE: Zinc Oxide Nanoparticles Based on Coffee Husks Embedded on Mesoporous Silica; [Co(5,5′-dmbipy)2(NCS)2]-SPE: Bipyridine Cobalt(ii) Complex-Modified Graphite Screen-Printed Electrode; 4HQ3CA/GC: Glassy Carbon Electrode Based on 4-Hydroxyquinoline-3-Carboxylic Acid; AuNP/NCNO: Gold Nanoparticles and Nitrogen-doped Carbon Nano-Onions.

**Table 3:** Determination of ACOP in Africure and Doliprane commercial capsules with GCE/ZnONPs-Sa/Phe.

Quantity	Africure	Doliprane
Initial mass of the packet (mg)	604	1088
Mass of the ACOP obtained after weighing (mg)	597	1039
Initial mass of the ACOP (mg)	500	1000
Theoretical mass of ACOP in the tested ACOP (mg)	494.205	957.598
Mass of ACOP determined by GCE/NPZnO-(Sa)/Phe (mg)	508.24	930.594
Recovery rate (%)	102.84	97.18

## Data Availability

The original contributions presented in this study are included in the article/Supplementary Material. Further inquiries can be directed to the corresponding authors.

## Supplementary Materials

The following supporting information can be downloaded at: https://www.mdpi.com/article/10.3390/bios16050244/s1, Figure S1: Effect of different interfering species on the peak currents of ACOP (ACOP:Interfering specie) in PB solution (pH = 2) on GCE/ZnONPs-Sa/Phe. The black curve is the blank, the red curve is the ratio 1:1, and the green curve is the ratio 1:2.
