# Supplementary material for: Development of an Electrochemical Platform Based on Zinc Oxide Nanoparticles Embedded onto Montmorillonite Clay Functionalized with Phenylalanine for the Nano-Sensing of Acetaminophen in Pharmaceutical Tablets
Source: Biosensors (Basel). 2026 Apr 26;16(5):244. doi: 10.3390/bios16050244 (PMC13204949; doi:10.3390/bios16050244)
Supplement: Supplementary file 1 [file biosensors-16-00244-s001.zip › biosensors-4231285-supplementary.pdf]

Supporting information

1. Interference study of ZnONPs-Sa/Phe/GCE

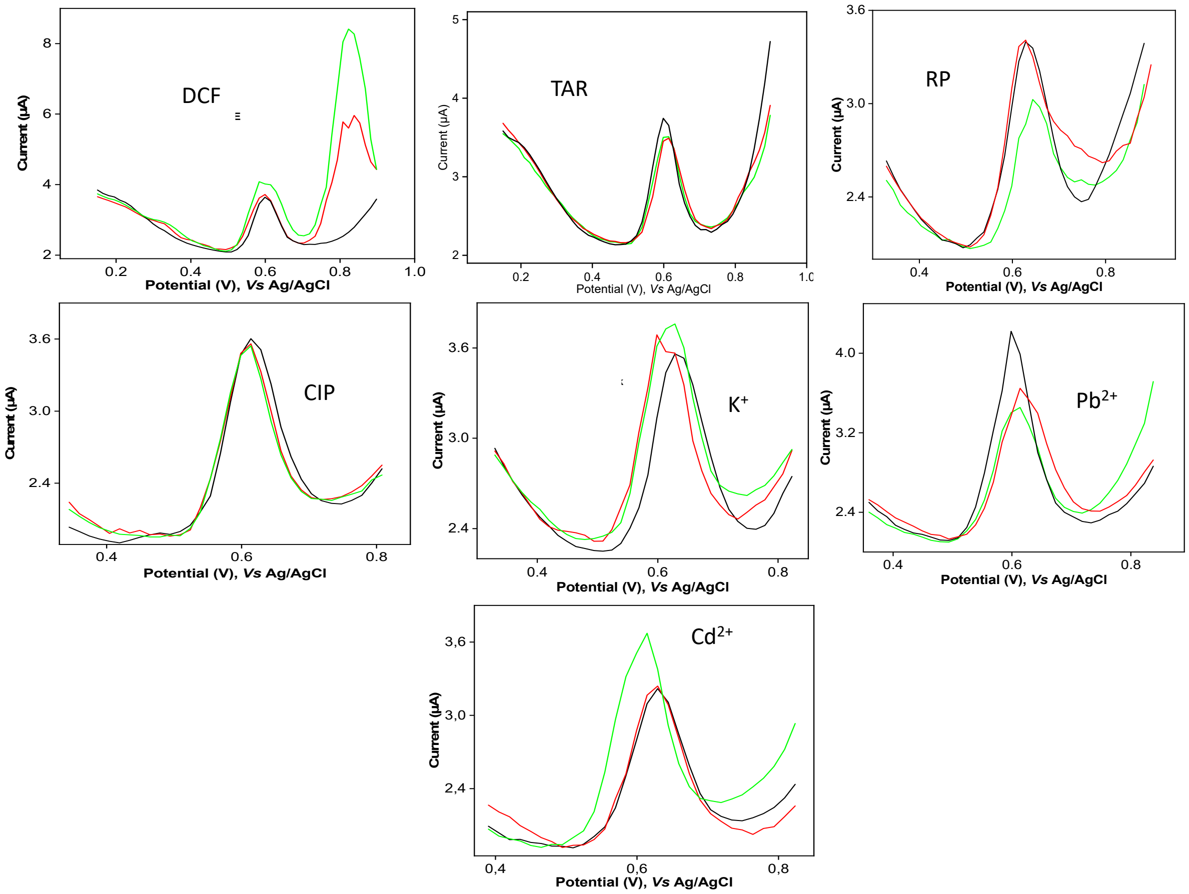

Figure S1: Effect of different interfering species on the peak currents of ACOP (ACOP:Interfering specie) in PB solution (pH = 2) on GCE/ZnONPs-Sa/Phe. The black curve is the blank, the red curve is the ratio 1:1, and the green curve is the ratio 1:2.
